# Integrated Role of *Bifidobacterium animalis* subsp. *lactis* Supplementation in Gut Microbiota, Immunity, and Metabolism of Infant Rhesus Monkeys

**DOI:** 10.1128/mSystems.00128-16

**Published:** 2016-11-29

**Authors:** Xuan He, Carolyn M. Slupsky, James W. Dekker, Neill W. Haggarty, Bo Lönnerdal

**Affiliations:** a Department of Nutrition, University of California, Davis, Davis, California, USA; b Department of Food Science and Technology, University of California, Davis, Davis, California, USA; c Fonterra Research and Development Centre, Fonterra Co-operative Group, Palmerston North, New Zealand; University of California San Diego

**Keywords:** infant, metabolome, microbiome, nutrition, probiotics

## Abstract

To investigate the impact of probiotic supplementation of infant formula on immune parameters, intestinal microbiota, and metabolism, five individually housed infant rhesus monkeys exclusively fed standard infant formula supplemented with probiotics (*Bifidobacterium animalis* subsp. *lactis* HN019) from birth until 3 months of age were compared with five standard formula-fed and five breast-fed monkeys. Anthropometric measurements, serum insulin, immune parameters, fecal microbiota, and metabolic profiles of serum, urine, and feces were evaluated. Consumption of *B. lactis*-supplemented formula reduced microbial diversity, restructured the fecal microbial community, and altered the fecal metabolome at the last two time points, in addition to increasing short-chain fatty acids in serum and urine. Circulating CCL22 was lower and threonine, branched-chain amino acids, urea, and allantoin, as well as dimethylglycine in serum and urine, were increased in the group supplemented with *B. lactis* compared with the standard formula-fed group. These results support a role of probiotics as effectors of gut microbial activity regulating amino acid utilization and nitrogen cycling. Future risk-benefit analyses are still needed to consolidate the existing knowledge on the long-term consequences of probiotic administration during infancy.

**IMPORTANCE** Probiotics are becoming increasingly popular due to their perceived effects on health, despite a lack of mechanistic information on how they impart these benefits. Infant formula and complementary foods are common targets for supplementation with probiotics. However, different probiotic strains have different properties, and there is a lack of data on long-term health effects on the consumer. Given the increasing interest in supplementation with probiotics and the fact that the gastrointestinal tracts of infants are still immature, we sought to determine whether consumption of infant formula containing the probiotic *Bifidobacterium animalis* subsp. *lactis* HN019 for 3 months starting at birth would impact gut microbial colonization, as well as infant immunity and metabolism, when compared with consumption of formula alone.

## Abstract (precis)

Probiotics are becoming increasingly popular due to their perceived effects on health, despite a lack of mechanistic information on how they impart these benefits. Infant formula and complementary foods are common targets for supplementation with probiotics. However, different probiotic strains have different properties, and there is a lack of data on long-term health effects on the consumer. Given the increasing interest in supplementation with probiotics and the fact that the gastrointestinal tracts of infants are still immature, we sought to determine whether consumption of infant formula containing the probiotic *Bifidobacterium animalis* subsp. *lactis* HN019 for 3 months starting at birth would impact gut microbial colonization, as well as infant immunity and metabolism, when compared with consumption of formula alone.

## INTRODUCTION

Early diet is a critical factor that shapes immune function, metabolism, and gut microbiota of an infant (1). In particular, gut microbiota serves as an important link between diet and health due to its ability to regulate energy homeostasis through interactions with the host epithelium and immune cells (2, 3). Gut microbiota is thus hypothesized to contribute to a programming effect that may impact metabolic phenotype in adulthood. One approach to modulate the functional capability of indigenous intestinal microbiota is through oral administration of probiotics (1, 4).

Beyond the rationale of probiotic application and its attempts to enhance health by altering the gut microbiota, there is considerable discussion about the benefits and risks related to the administration of formula supplemented with probiotics, particularly in very young infants (5, 6). Indeed, there are still too many uncertainties to draw unequivocal conclusions due to the complex nature of the host condition (healthy versus specific illnesses or disorders), timing (age), duration of treatment, dosage, form of food matrix (infant formula, follow-on formula, or special medical foods), strain specificity, and viability of the probiotic (5). Furthermore, multiple health targets and unspecified definitions of what is harmful or beneficial raise the challenge of drawing a sole conclusion on probiotic use in infancy. Therefore, a molecular-level approach will result in a better understanding of the underlying mechanisms.

*Bifidobacterium lactis* sp. is more oxygen tolerant than other *Bifidobacterium* strains (7). It was first isolated from fermented milk samples and recently was identified in feces from human infants, rabbits, and chickens, as well as in sewage (8). More recently, *Bifidobacterium lactis* sp. has been regrouped as *Bifidobacterium animalis* subsp. *lactis*. One strain, HN019, was first isolated from yogurt and documented with a unique capability to survive at low pH and in the presence of bile acids (9), making it a promising candidate for probiotic use.

The strain HN019 is nontoxic to mice (10–12) and has been reported to be well tolerated in human infants (13). However, infancy is characterized by microbial plasticity, and limited data on the clinical effectiveness as well as microbiological and metabolic consequences of adding probiotic preparations to infant formula have not been detailed. Here, we use the infant rhesus monkey as a model to understand the influence of *Bifidobacterium animalis* subsp. *lactis* HN019 (*B. lactis*)-supplemented infant formula on gut microbiota, immunity, and metabolism in comparison to standard formula feeding or breast-feeding. Our study serves as an extensive validation model for clinical observation and provides a systemic view of dietary response to probiotic intervention during infancy.

## RESULTS

We have previously published the metabolic impacts of formula feeding on a model of human infant feeding, the infant rhesus macaque model (1). Here, we extend this study to examine how the addition of the probiotic *Bifidobacterium animalis* subsp. *lactis* strain HN019 in infant formula affects host immune function, microbial succession, and metabolism.

### Growth trajectory and immune response.

**FIG 1:**
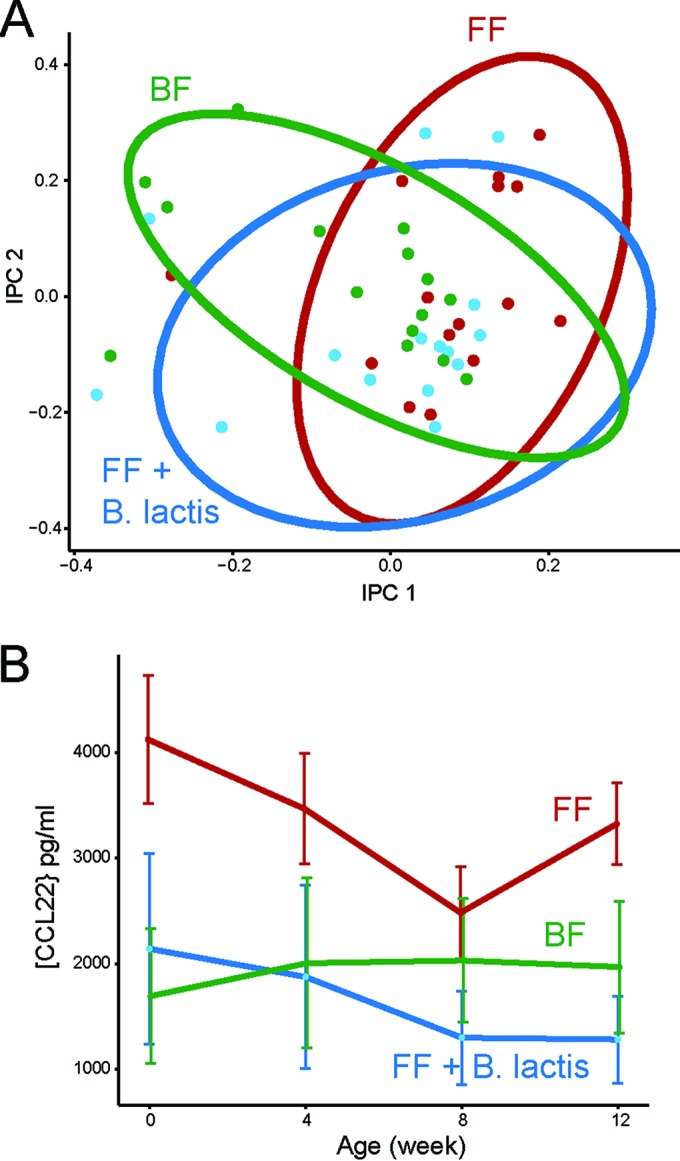
Effect on immune response following *B. lactis* administration. (A) Independent principal-component analysis (iPCA) score plot of immune functional data of infant rhesus macaques strictly breast-fed (BF, green), fed standard infant formula (FF, red), or fed infant formula supplemented with *B. lactis* (FF + *B*. *lactis*, blue). The ellipses were constructed based on multivariate normal distribution at 95% confidence interval. (B) Serum CCL22 concentration (mean ± SEM) was significantly higher in formula-fed infant rhesus monkeys than in the *B. lactis*-supplemented infant formula group (rmANCOVA, *P* < 0.05 after FDR adjustment).

In our previous study, we showed increased weight gain and crown-rump length (CR) measurements as well as elevated serum insulin levels in formula-fed infant monkeys compared with their breast-fed counterparts. Here, we observed that addition of *B. lactis* to infant formula did not impact formula intake, weight, intake over weight, or CR length overall (repeated-measures analysis of variance [rmANOVA]) or at each individual time point (multiple *t* tests) compared with their standard formula-fed counterparts (data not shown). This suggests that supplementation with *B. lactis* did not cause the infant-formula-derived phenotypic state to be more similar to the breast-fed state. Furthermore, there was no probiotic-related adverse event observed throughout the study, which suggests that *B. lactis* was well tolerated by rhesus infant macaques. Immune function analysis using a panel of cytokines and growth factors revealed similar profiles between the infants fed *B. lactis*-supplemented formula and those fed standard formula (Fig. 1A). Interestingly, we found a significant decrease of CCL22 (repeated-measures analysis of covariance [rmANCOVA], *P* < 0.05 after false discovery rate [FDR] adjustment) for the *B. lactis*-supplemented group compared to the standard formula group but no significant difference compared to the breast-fed group (Fig. 1B).

### Gut microbial metabolism and community.

**FIG 2:**
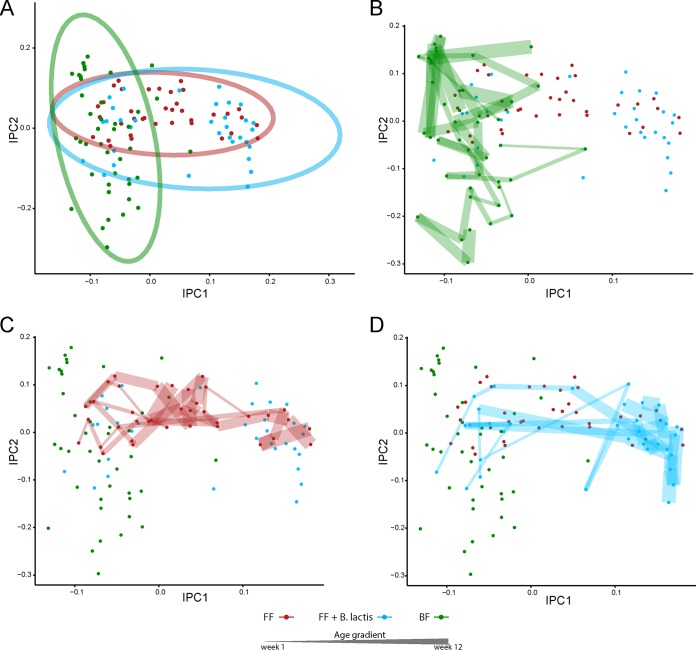
^1^H NMR-based fecal metabolomics assessment of gut microbial functional activity in response to monkey milk, standard infant formula, or *B. lactis*-supplemented infant formula. (A) Score plot from independent principal-component analysis (iPCA) of fecal metabolic profiles of infant rhesus macaques fed breast milk (BF, green), infant formula (FF, red), or infant formula supplemented with *B. lactis* (FF + *B. lactis*, blue). Ellipses were constructed based on multivariate normal distribution at 95% confidence interval. (B, C, and D) Multiple observations from the same rhesus monkey infant were age ascendingly linked by lines and presented as increasing thickness with age.

One of the impacts of formula feeding on host metabolism and physiology is through shifting of the gut microbial community structure. In the present study, we first examined the fecal metabolomics profiles of breast-fed and formula-fed infant rhesus macaques and compared them with infant macaques fed formula supplemented with *B. lactis* to determine if functional changes could be observed. The independent principal-component analysis (iPCA) reveals a difference between the fecal metabolic profiles of breast-fed and formula-fed infants (Fig. 2A). Interestingly, following the developmental pattern of the microbiota over time revealed profound dynamic shifts (Fig. 2B to D). Although supplementation with *B. lactis* in infant formula was unable to counter the dynamic nature of, and the background noise from, the infant gut microbial succession, differences between infants fed standard formula and infants fed *B. lactis*-supplemented formula became apparent in later weeks (Fig. 2C and D).

**FIG 3:**
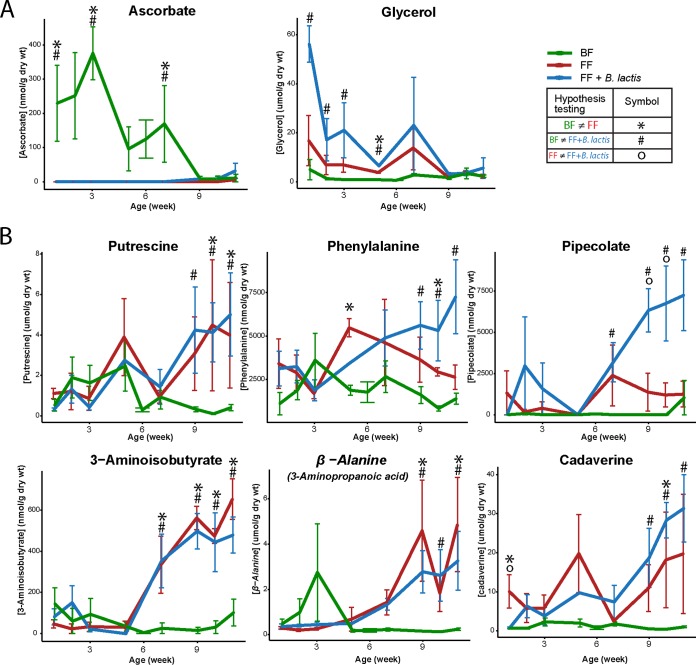
Comparison of fecal metabolite concentrations in breast-fed (BF, green), standard formula-fed (FF, red), and *B. lactis*-supplemented formula-fed (FF + *B. lactis*, blue) infant rhesus monkeys from birth to 3 months of age. Fecal metabolites that were significantly altered were grouped by their responding pattern over time as either early-onset markers (A) or delayed-response markers (B). The significant difference at each time point was evaluated using one-way ANOVA followed by *post hoc* Tukey test (*P* < 0.05). Data are presented as means ± SEM.

Throughout the 12-week-long study, a breast-feeding-driven fecal metabolic marker exhibiting a pattern that was either consistently increased or decreased over all time points compared to the formula-fed counterparts was not observed. Therefore, two sets of metabolites based on the characteristics of being either significantly different at earlier time points (“early-onset markers”) or significantly different at later time points (“delayed-response markers”) were defined (Fig. 3). Lactate was significantly higher in the *B. lactis*-supplemented group than in the breast-fed and standard formula-fed groups only in the first week (first observational time point) and not different at other time points (one-way ANOVA followed by *post hoc* test; breast-fed, 150.0 ± 37.8; standard formula fed, 2,334.0 ± 4,450.3; and *B. lactis*-supplemented formula fed, 14,673.6 ± 13,635.1 nmol/g [dry weight], expressed as mean ± standard deviation). In the first 2 months, fecal ascorbate was significantly higher in the breast-fed infants, whereas glycerol was elevated in the *B. lactis*-supplemented group. In the 3rd month of life, the two infant formula groups shifted to a different trajectory, which was driven by elevated putrescine, phenylalanine, pipecolate (higher only in the *B. lactis* group), 3-aminoisobutyrate, β-alanine, and cadaverine compared to their breast-fed counterparts (Fig. 3, one-way ANOVA followed by *post hoc* test). Overall, individual short-chain fatty acids (SCFA) were observed to be highly dynamic over time but were not significantly different between groups (rmANOVA; see Fig. S1 in the supplemental material).

10.1128/mSystems.00128-16.1 Figure S1  Mean fecal short-chain fatty acid concentrations for the standard formula-fed (FF), *B. lactis*-supplemented formula-fed (FF + *B. lactis*), and breast-fed (BF) groups. Download Figure S1, TIF file, 0.8 MB. Copyright © 2016 He et al. 2016 He et al. This content is distributed under the terms of the Creative Commons Attribution 4.0 International license.

**FIG 4:**
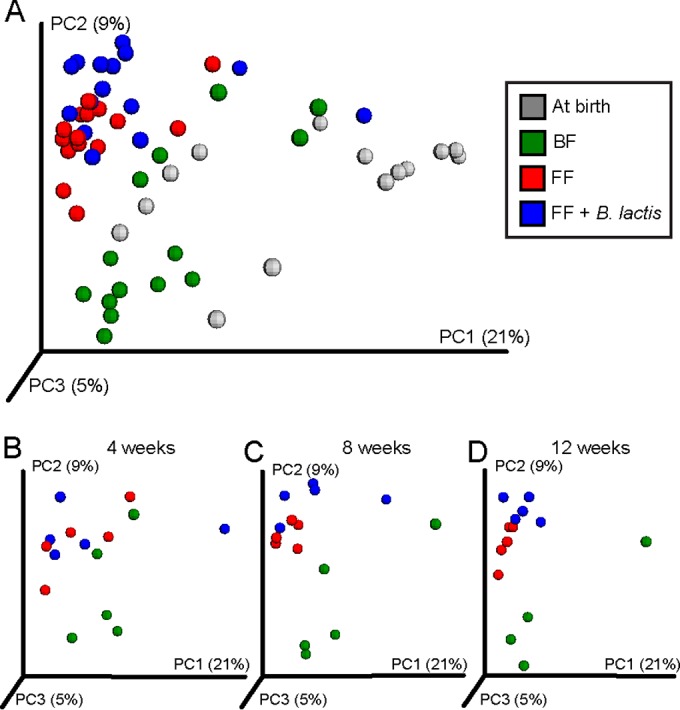
16S rRNA gene survey reveals a moderate impact of formula-based *B. lactis* supplementation on the fecal microbial community profile. (A) Principal-coordinate analysis (PCoA) of unweighted UniFrac distance of 16S rRNA gene sequences exhibits a difference between the breast-fed group and the two formula-fed groups. (B to D) The corresponding change at 4 weeks (B), 8 weeks (C), and 12 weeks (D) of age between the two formula-fed groups suggested a shift in fecal microbial community structure in response to *B. lactis* supplementation that was moderate and temporal at the time of observation, exhibiting a trend of moving toward a state that was different from that of the standard infant formula group near the end of the study.

Differences in gut microbiota due to *B. lactis* supplementation were reflected as differences in 16S rRNA microbial community profiles (Fig. 4). Analysis and comparison of the fecal microbiome across the standard formula-fed, *B. lactis*-supplemented formula-fed, and breast-fed groups suggest that both formula groups developed similarly and were different from the breast-fed group, especially at weeks 8 and 12. Moreover, each of the formula groups tended to cluster tightly together at 12 weeks compared with previous weeks, illustrating subtle differences in the microbiome with *B. lactis* supplementation. Importantly, the direction of the *B. lactis*-induced shift was not to a state more similar to the breast-fed group. Overall, this fecal microbiome result is in agreement with the fecal metabolomics data, suggesting that the effects of *B. lactis* on the microbial community are not immediate but develop over time.

**FIG 5:**
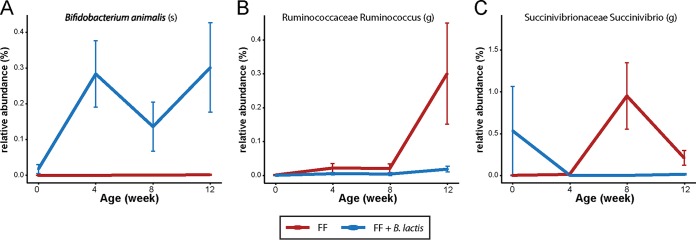
Plot of relative abundance of *Bifidobacterium animalis* (A), *Ruminococcus* (*Ruminococcaceae*) (B), and *Succinivibrio* (*Succinivibrionaceae*) (C) (repeated-measures ANOVA without multiple-comparison correction, at *P* = 0.0001, *P* = 0.02, and *P* = 0.01, respectively). Data are presented as means ± SEM.

Further examination of the microbial α diversity revealed a significant reduction of observed species in the *B. lactis*-supplemented monkeys compared with those fed standard infant formula at both 8 and 12 weeks of age but not at birth and week 4 (*t* test, *P* < 0.05). Interestingly, the presence of *B. animalis* in feces was detected only after *B. animalis* subsp. *lactis* strain HN019 supplementation and contributed only about 0.2 to 0.4% of the total microbial population (Fig. 5A). Moreover, the relative abundance of bifidobacteria in feces was not significantly affected by *B. lactis* supplementation compared with the standard formula group, suggesting that *B. lactis* did not compete for resources or cross-feed other bifidobacterial species. Interestingly, *B. lactis* supplementation slightly reduced the abundance of the genera *Ruminococcus* (from the *Ruminococcaceae* family) and *Succinivibrio* compared to the standard formula group (confirmed by both rmANOVA and rmANCOVA; *P* < 0.05 before FDR adjustment [Fig. 5B and C]).

### Metabolism.

**FIG 6:**
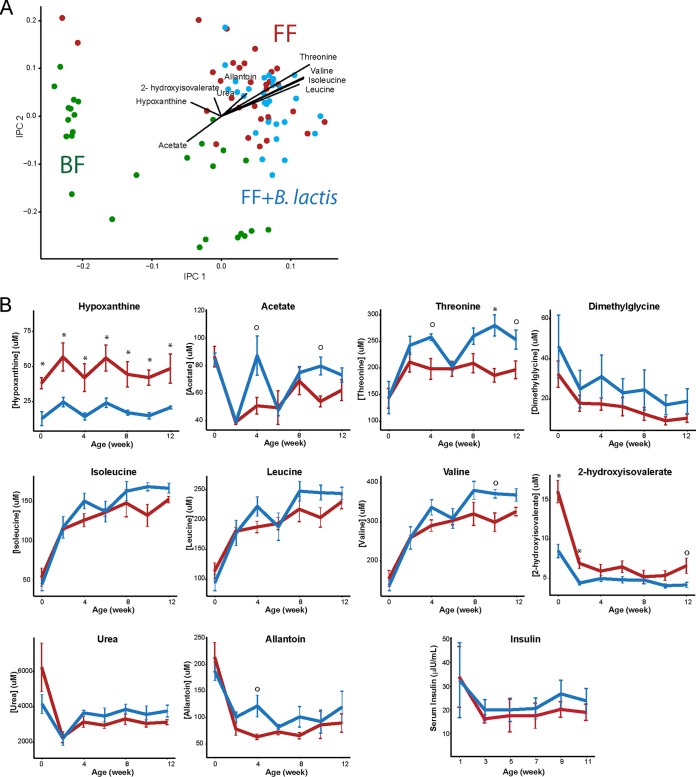
^1^H NMR-based serum metabolomics revealed minor differences between standard formula-fed and *B. lactis*-supplemented formula-fed rhesus infants. Serum hypoxanthine, acetate, and threonine levels were significantly different (*P* = 7E−05, *P* = 0.03, and *P* = 0.001, respectively), as were isoleucine, valine, and allantoin levels (*P* = 0.04, *P* = 0.03, and *P* = 0.02, respectively). Dimethylglycine, leucine, urea, and insulin levels were not significantly different (*P* = 0.1, *P* = 0.1, *P* = 0.1, and *P* = 0.5, respectively). Data are presented as means ± SEM, and overall *P* values were determined using rmANCOVA without multiple-comparison correction. Differences at specific time points were determined using independent-sample *t* tests and are indicated as * (*P* < 0.05 after FDR adjustment) and ○ (*P* < 0.05 before FDR adjustment). Data are presented as means ± SEM.

Examination of the overall serum metabolic profile of *B. lactis*-supplemented monkeys did not reveal a clear difference compared to those who consumed standard infant formula (Fig. 6A). Univariate analyses revealed that monkeys consuming *B. lactis*-supplemented formula experienced higher levels of circulating acetate, hypoxanthine, threonine, branched-chain amino acids (BCAA; isoleucine and valine), 2-hydroxyisovalerate, and allantoin (rmANCOVA, *P* < 0.05 [Fig. 6B]). Urea, leucine, and dimethylglycine were moderately elevated in the *B. lactis*-supplemented formula-fed group compared to the group fed standard formula (rmANCOVA, *P* < 0.1). The corresponding *t* test for each metabolite further confirms the above result and unveils the consistent reduction of hypoxanthine in the *B. lactis*-supplemented group, whereas changes in acetate, valine, and threonine were more profound from week 4 onward (Fig. 6B). Furthermore, the elevated amino acids in the *B. lactis* group were not coupled with a significant increase in insulin concentration compared to those consuming standard infant formula (Fig. 6B).

**FIG 7:**
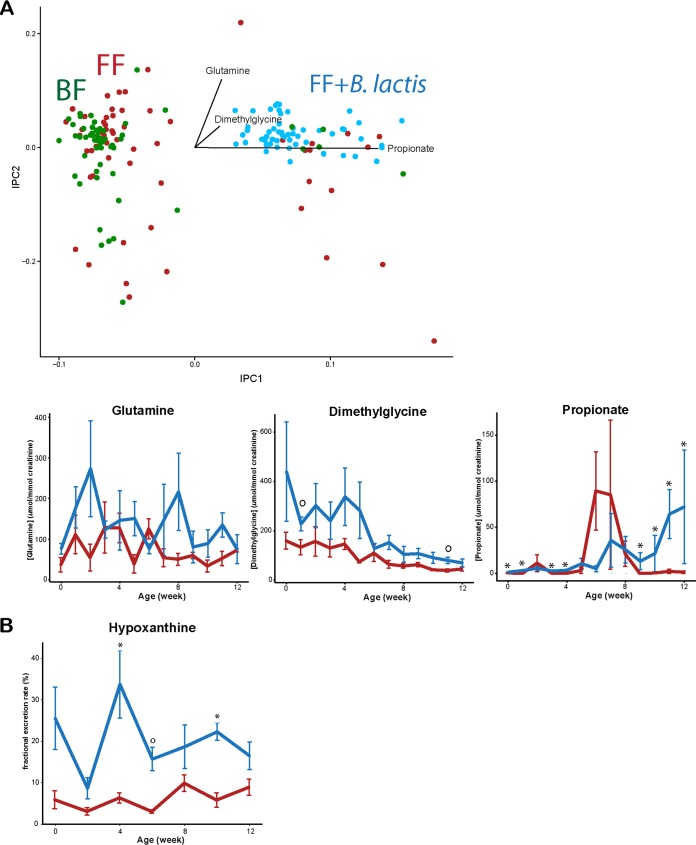
^1^H NMR-based urinary metabolomics assessment revealed different profiles between standard formula-fed and *B. lactis*-supplemented formula-fed rhesus infants. (A) Urinary glutamine, dimethylglycine, and propionate levels were significantly higher in the *B. lactis*-supplemented group (rmANCOVA without multiple-comparison correction at *P* = 0.03, *P* = 0.004, and *P* = 0.0002, respectively). (B) Fractional excretion rate of hypoxanthine was significantly higher in the *B. lactis*-supplemented formula-fed rhesus infants (rmANCOVA without multiple-comparison correction, *P* = 0.001). Fraction excretion of hypoxanthine was defined as [urine hypoxanthine] × [serum creatinine]/[serum hypoxanthine]/[urine creatinine]. Differences at specific time points were determined using independent-sample *t* tests and are indicated as * (*P* < 0.05 after FDR adjustment) and ○ (*P* < 0.05 before FDR adjustment). Data are presented as means ± SEM.

The urine metabolome of *B. lactis*-supplemented monkeys revealed a profound difference from both the breast-fed and standard formula-fed monkey infants (Fig. 7A). In particular, urinary glutamine and dimethylglycine were consistently higher in the monkeys fed *B. lactis*-supplemented formula, whereas the urinary propionate level was elevated starting from 7 weeks until the end of the experiment (rmANCOVA, *P* < 0.05). However, except for propionate, the corresponding *t* test at each week showed a weak significant difference (Fig. 7A).

We further investigated the serum and urine data by determining the fractional excretion rate of each metabolite. The only significant difference in fractional excretion rate was found for hypoxanthine, which was significantly higher in the monkeys fed *B. lactis*-supplemented formula (rmANCOVA, *P* < 0.05; also confirmed by multiple *t* tests at each time point). These results suggest that the decrease of hypoxanthine in serum is likely due to a higher excretion rate of hypoxanthine in the urine (Fig. 7B).

## DISCUSSION

We have previously reported on the metabolic impacts of formula feeding in a model of human infant feeding, the infant rhesus macaque model (1). Rhesus macaques were chosen for this study due to their similarity to humans in terms of genetics and the metabolome (14). Indeed, it has been previously shown that infant rhesus macaques can easily tolerate human infant formula (15, 16), as it is regularly used in primate nurseries. Our previous work highlighted differences in the microbiome, immune function, and metabolism between breast-fed and formula-fed monkeys (1). Here, we extend this study to examine how addition of the probiotic *Bifidobacterium animalis* subsp. *lactis* strain HN019 affects the gut microbiota as well as host immune function and metabolism.

As was reported previously (13), no adverse effects on intake or growth parameters were observed when *B. lactis* was consumed with standard infant formula. Except at birth, for all weeks *B. lactis* was detected in the feces; however, whether *B. lactis* can colonize the infant gut after supplementation is stopped was not examined in this study. These data indicate that early-life supplementation with *B. lactis* results in an elevation of short-chain fatty acids (SCFA) in serum (acetate) and urine (propionate), but not in feces, without extensively shifting the overall fecal microbiota composition. In a previous study, an investigation of supplementation with 3 g of probiotics containing the bacterial strains *Bifidobacterium bifidum* W23, *B*. *animalis* subsp. *lactis* W52, and *Lactococcus lactis* W58 given to infants at the age of 3 months resulted in elevated fecal SCFA (acetate, butyrate, propionate, and isobutyrate) production (17), whereas a combination of *Lactobacillus acidophilus* 74-2 and *B. animalis* subsp. *lactis* DGCC 420 had no effect on fecal SCFA in adults (18). Interestingly, a microbiological study on *B. lactis* culture medium suggested that *B. lactis* can produce only acetate, lactate, and formate but not propionate (7); therefore, we speculate that there could be increased cross-talk between *B. lactis* and other gut microbes.

The effect of *B. lactis* supplementation on host metabolism may be mediated by an interaction with the gut microbiota community. Indeed, a reduction in microbial diversity as well as a minor effect on relative abundance of *Ruminococcus* (from the *Ruminococcaceae* family) and *Succinivibrio* in response to *B. lactis* supplementation was observed. Several studies have demonstrated a role for this strain (HN019) in regulating other bacteria and aiding in resistance to intestinal infections. The HN019 strain has been shown to inhibit attachment of *Salmonella enterica* serovar Typhimurium to INT-407 cells (19). Culture medium from HN019 was shown to be lethal to *Escherichia coli* (20), and consumption of this strain was shown to improve survival in mice challenged with *Salmonella* (21), as well as to decrease diarrhea and rotavirus load and increase IgA, IgM, and IgG for rotavirus and *E. coli* in piglets (22). Moreover, mice fed HN019 1 week prior to oral challenge with *E. coli* exhibited reduced severity of infection and improved immune markers (23).

Changes in gut microbial composition and functional activity may also correspond to an alteration in immune response. In the present study, most of the investigated immunological parameters were not influenced by *B. lactis* supplementation, except for CCL22, which was lower in the *B. lactis*-supplemented group than in the standard formula group, with a level similar to that of their breast-fed counterparts. CCL22 is important for attracting CCR4 receptor-expressing cells, including Th2 lymphocytes, mast cells, dendritic cells, and natural killer T lymphocytes, to sites of inflammation (24) and thus is considered important in development of the allergic response. Interestingly, it has been shown that the only chemokines measured in cord blood that are predictive of future allergy are CCL22 and CCL17 (25). CCL22 is higher in those who experience allergic sensitization later in life, and increased CCL17 is associated with allergic symptoms (25). Furthermore, in a separate study, administration of a probiotic mixture containing *B. bifidum* W23, *B*. *animalis* subsp. *lactis* W52, and *Lactococcus lactis* W58 reduced the incidence of eczema in infants in their first 3 months of life (26). A follow-up study of the fecal metabolome of these children showed a significantly higher level of SCFA in the feces of those who later developed eczema (17). Combined with our results, these findings support the notion that probiotic supplementation during infancy may be effective in influencing intestinal microbiota and potentially prevent future allergy.

Although probiotics have been proposed to promote gut health, studies on infants are limited. Introduction of cereals with addition of *Lactobacillus paracasei* subsp. *paracasei* F19 to infants from 4 to 13 months of age revealed no significant difference in the overall plasma metabolic profile but a significant elevation of plasma tryptophan and putrescine in the probiotic-supplemented group at 13 months of age (27). In the present study, we also observed a significant increase in serum tryptophan. Furthermore, branched-chain amino acids (BCAA; leucine, isoleucine, and valine) and nitrogen waste products (urea and allantoin) showed a trend toward elevated levels in the monkeys fed the *B. lactis*-supplemented formula (Fig. 6). Since *B. lactis* was the only constituent that was modified in the diet, we speculate that alterations in amino acid metabolism may have a gut microbial origin. Interestingly, it has previously been shown in infants that circulating lysine can be derived from oral introduction of urea (28). In addition, the human distal gut microbiota harbors genes enriched in KEGG pathways of lysine biosynthesis and phenylalanine, tyrosine, and tryptophan biosynthesis, as well as BCAA biosynthesis (29, 30). These microbially derived amino acids could be precursors for SCFA synthesis and contribute to the host amino acid pool (reviewed in reference 31). These results support the role of probiotics in modulating gut microbiota community structure and functional activity to regulate amino acid utilization and nitrogen cycling.

We observed increased dimethylglycine in the urine and serum of monkeys fed *B. lactis*-supplemented formula in comparison to formula-fed and breast-fed infant monkeys. Dimethylglycine is a component of betaine-homocysteine metabolism and specifically part of the methionine cycle responsible for generation of reactive oxygen species (ROS) (32). To the best of our knowledge, there are only two metabolomics-based animal studies where rodents were supplemented with probiotics (33, 34). Both studies observed such a change in dimethylglycine in response to probiotic supplementation, suggesting an underlying mechanism that is related to the transmethylation metabolic pathway (homocysteine-betaine) that closely interconnects with phosphocholine, choline, betaine, dimethylglycine, sarcosine, betaine, and formation of methionine from homocysteine in the liver and pancreas. Martin et al. (34) showed elevation of hepatic dimethylglycine in mice fed with *Lactobacillus rhamnosus* and those fed with *L. rhamnosus* and a prebiotic galacto-oligosaccharide in their diet. Furthermore, the change in hepatic dimethylglycine was negatively correlated with lipid concentration in the kidney cortex. Probiotics alone did not induce a significant difference in pancreatic dimethylglycine levels; however, consumption of the symbiotic diet was correlated with a higher level of dimethylglycine in the pancreas (34). Similarly, administration of *Lactobacillus acidophilus* La5 and *Bifidobacterium lactis* Bb12 resulted in higher urinary *N*-methylnicotinamide, dimethylglycine, and choline than in control rats (33). Our results are consistent with the findings of previous studies, supporting the notion that probiotic supplementation may have an impact on betaine-homocysteine metabolism.

Hypoxanthine was decreased in the serum of the *B. lactis*-supplemented group and was coupled with an increased urinary hypoxanthine clearance rate. At present, we are unable to explain the underlying mechanism for the change in hypoxanthine, but one possibility may be that purine metabolism is also affected due to excess bacterial nucleotides from the probiotic supplement. Xanthine oxidase, an enzyme that catalyzes the oxidation of hypoxanthine to xanthine, could generate ROS, leading to oxidative stress (35). However, changes in the activity or expression of enzymes involved in response to *B. lactis* supplementation still remain to be determined, and biological mechanisms responsible for this increase in hypoxanthine level will need to be elucidated.

Our results support the hypothesis that probiotic use in early life can modulate the development of an infant’s immune system and gut microbiota, which in turn is reflected as changes in the metabolome. However, the magnitude of the change did not override the effect of the formula, as infant monkeys consuming *B. lactis*-supplemented formula showed a greater similarity to those consuming standard formula than to those being breast-fed. By comparison, the metabolomics data revealed a much greater change in response to *B. lactis* supplementation than the immune or microbiome data, demonstrating a broad efficacy of metabolomics-based applications as an effective discovery approach. The molecular and functional integration of immune, gut microbiota and metabolic responses provides a framework that reveals systemic alterations in response to diet during infancy, allowing for discovery of biomarkers that could be used to inform the impact of food and nutrition on infant health. Despite the growing popularity of probiotic supplementation of infant formula, it is difficult to generalize this result, as the effects of probiotics on immune response, gut microbiota, and metabolism are highly strain specific, dosage specific, and likely age and diet dependent. Our data help to elucidate the molecular basis underlying probiotic exposures during infancy, but the long-term consequences remain to be investigated.

## MATERIALS AND METHODS

### Animals and experimental diets.

Five individually housed infant rhesus monkeys (*Macaca mulatta*; 3 males and 2 females) were exclusively fed a standard infant formula supplemented with *Bifidobacterium animalis* subsp. *lactis* HN019 (7 × 10^5^ CFU/ml or 5 × 10^6^ CFU/g infant formula) from birth until 3 months of age and compared with rhesus infants fed standard formula and breast-fed infants described in our previous infant-feeding study (1). The standard infant formula (described elsewhere [1]) and the *B. lactis* supplement were kindly provided by Fonterra Research and Development Centre (Fonterra Co-operative Group, Palmerston North, New Zealand). Formula containing *B. lactis* was prepared fresh by animal care staff by suspension of the freeze-dried *B. lactis* powder in filtered water, which was added to freshly prepared formula prior to each meal. Infants were fed according to an animal husbandry protocol that started with hand feeding every 2 h from a nursing bottle with a nipple for up to 5 days and progressed to self-feeding. Rhesus monkeys used for this study were maintained at the California National Primate Research Center, Davis, CA. Protocols were approved by the University of California, Davis Institutional Animal Care and Use Committee and conducted in accordance with the Department of Agriculture Animal Welfare Act.

### Sample collection.

Infant formula intake was recorded daily. Anthropometric measurements (weight and crown-rump length) were recorded every other week starting at birth. Urine samples were collected at birth and every week up to 12 weeks of age as described previously (1). Blood samples were drawn weekly in the morning via femoral venipuncture and divided such that metabolomics analysis was performed on samples collected every 2nd week from birth and serum insulin was analyzed every 2nd week from week 1. Animals were not subjected to fasting prior to blood collection. Blood samples were allowed to clot at room temperature for 30 min, followed by centrifugation. Fecal samples were collected weekly. Fecal samples at birth as well as at weeks 4, 8, and 12 were used for microbial profiling, and all remaining samples were used for fecal metabolomics. Once collected, urine, fecal, and serum samples were frozen at −20°C followed by long-term storage at −80°C until analysis. All individuals handling specimens were aware of all hazards associated with working with Old World nonhuman primates and as such adhered to standard biological safety level 2 procedures when handling samples.

### Fecal metabolite extraction.

To extract metabolites from stools of various dry weights, fecal samples (wet, 0.30 ± 0.19 g; dry, 0.06 ± 0.04 g) were prepared using the Bligh and Dyer method (36) with some modifications. First, dried fecal samples were thoroughly diluted with 4 volumes of ultrapure water, followed by mixing with 4 volumes of methanol-chloroform (2:1, vol/vol) containing 0.5 mg/ml butylated hydroxytoluene (Sigma, St. Louis, MO). Samples were subsequently mixed with 1 volume of chloroform and finally 1 volume of ultrapure water. Samples were vortexed for 15 s three times between each addition. After standing for 10 min at room temperature, samples were centrifuged at 70 × *g* for 15 min, and the top methanol-water layer was carefully collected, measured, and mixed with 4 volumes of ultrapure water. Methanol was removed using a Genevac miVac Duo concentrator (Stone Ridge, NY), and samples were subsequently lyophilized using a Labconco FreeZone 4.5-liter freeze-dry system (Labconco, Kansas City, MO). Samples were stored at −80°C until sample preparation.

### Immune function parameter, insulin, microbiome, and metabolomics analysis.

Experimental procedures were similar to what was described previously (1). Briefly, serum cytokines were analyzed using the cytokine monkey magnetic 28-plex panel for quantitative analysis of epidermal growth factor (EGF), fibroblast growth factor basic (FGF-basic), hepatocyte growth factor (HGF), vascular endothelial growth factor (VEGF), granulocyte colony-stimulating factor (G-CSF), granulocyte-macrophage colony-stimulating factor (GM-CSF), macrophage migration inhibitory factor (MIF), tumor necrosis factor alpha (TNF-α), eotaxin, interferon gamma (IFN-γ), interleukin-1β (IL-1β), interleukin-1 receptor antagonist (IL-1RA), IL-2, IL-4, IL-5, IL-6, IL-8, IL-10, IL-12, IL-15, IL-17, CCL2 (monocyte chemoattractant protein 1 [MCP-1]), CCL3 (macrophage inflammatory protein 1α [MIP-1α]), CCL4 (MIP-1β), CCL5 (RANTES), CCL22 (MDC), CXCL9 (MIG), and CXCL11 (I-TAC). Serum insulin was analyzed using a commercially available kit (Linco, St. Louis, MO) that was validated for cross-reactivity with nonhuman primate insulin.

Urine and serum samples for metabolomics were prepared as previously described (1). Fecal extracts were diluted in 260 to 340 μl of phosphate buffer (4.7 mM K_2_HPO_4_, 5.3 mM KH_2_PO_4_, pH 6.8) followed by centrifugation (14,000 × *g*, 10 min, 4°C). Serum samples were filtered through a 3,000-molecular-weight (MW)-cutoff filter to remove insoluble lipid particles and proteins. To 585 μl of serum filtrate, urine, or fecal extract, 65 μl of a solution consisting of 5 mM 3-(trimethylsilyl)-1-propanesulfonic acid-*d*6 (DSS-*d*6) and 0.2% NaN_3_ (bactericide) in 99.8% D_2_O was added both to act as an internal standard (DSS-*d*6) and to serve as nuclear magnetic resonance (NMR) lock solvent (D_2_O). The pH of each sample was adjusted to 6.8 ± 0.1 by adding small amounts of NaOH or HCl to minimize pH-sensitive peak shift. Six-hundred-microliter aliquots were transferred to 5-mm Bruker NMR tubes and stored at 4°C until NMR acquisition (within 24 h of sample preparation). NMR spectra were acquired using a Bruker Avance 600-MHz NMR spectrometer equipped with a SampleJet autosampler using a nuclear Overhauser effect spectroscopy (NOESY)-presaturation pulse sequence (noesypr) at 25°C with water saturation during the prescan delay (2.5 s) and mixing time (100 ms). A total of 32 transients over a spectral window of 12 ppm were acquired with an acquisition time of 2.5 s. Once acquired, all spectra were processed as previously described (1) using Chenomx NMR suite v8.1 (Chenomx Inc., Edmonton, Canada). Metabolite quantitation was achieved using the 600-MHz library as described previously (1, 37, 38). Serum concentrations are reported as micromolar concentrations. Urine concentrations were scaled relative to creatinine and expressed as micromoles per millimole of creatinine. Fecal samples were scaled based on dry weight and are presented as micromoles per gram (dry weight) or nanomoles per gram (dry weight).

### Microbiome analysis.

DNA was extracted from monkey stool samples as described previously (1). Briefly, samples were reconstituted in ice-cold phosphate-buffered saline to a final volume of 2 ml and centrifuged at 8,000 × *g* for 5 min. The pellet was suspended in 200 μl of lysis buffer (20 mM Tris-HCl [pH 8], 2 mM EDTA, 1.2% Triton X-100) containing 40 mg/ml of lysozyme (Sigma, St. Louis, MO) and incubated at 37°C for 30 min. DNA was extracted using the QIAamp DNA mini-stool kit (Qiagen Inc., Valencia, CA), according to the manufacturer’s directions with the addition of a bead beating step for 2 min after addition of the ASL buffer and heating for 5 min at 95°C. DNA extracts were stored at −80°C until further analysis. The V4 region of the 16S rRNA gene was amplified and purified using the procedure described previously (1) and subsequently submitted to the UC Davis Genome Center DNA Technologies Core for a 150-bp pair-end sequencing on an Illumina GAIIx platform.

The resulting sequences were analyzed in the Quantitative Insights Into Microbial Ecology (QIIME) pipeline (version 18.0) (39) using the close reference operational taxonomic unit (OTU) selection method. Briefly, OTU selection was performed using UCLUST (40) against the most recent version of the Greengenes core database (“gg_13_8_otus”) and clustered with a threshold of 97% identity, excluding reads that did not match the database. The data were refracted by the minimum observation account. Principal-coordinate analysis (PCoA) of the unweighted UniFrac distance (41) of 16S rRNA gene sequences was performed to illustrate the relationship between the between-sample (β) diversity values, and the result was visualized using Emperor (42). α diversity analysis was determined using the observed species function in QIIME and equivalent to the total count of unique OTUs found in the sample.

### Statistical analysis.

Prior to analysis, all metabolomics data suspected of contamination were removed, including urine samples contaminated with feces (samples with very high short-chain fatty acid concentrations) and fecal samples contaminated with food (high lactose) or urine (high urea and trimethylamine *N*-oxide). The fractional excretion rate of each metabolite was calculated as the product of [urine metabolite] × [serum creatinine]/[serum metabolite]/[urine creatinine]. To approximate normality, operational taxonomic unit (OTU) relative abundance was arcsine transformed, and all metabolomics and serum cytokine data were log_10_ transformed.

Independent principal-component analysis (iPCA) was performed to determine the differences between treatment groups on immune parameter measurements as well as the fecal, serum, and urine metabolite data using the R package mixomics (43). Data were mean centered, and the score plot as well as the variable correlations was visualized using ggplot2 (44). The ellipses were constructed based on multivariate normal distribution at 95% confidence levels.

To reveal the effect of *B. lactis* supplementation on age, body weight, crown-rump length (CR), formula intake, intake over weight, insulin, immune parameter measurements, OTU relative abundance, and metabolite concentration in feces, urine, and serum, a mixed-effects model repeated-measures ANOVA (rmANOVA) and repeated-measures ANCOVA (rmANCOVA) (for adjustment of baseline measurement [data collected at birth]) were used to identity age effects, diet effects, and age-by-diet interaction. Statistical differences at each time point were assessed using one-way ANOVA followed by *post hoc* Tukey test between three groups or independent sample *t* tests between two groups. All *P* values from multiple-comparison tests were adjusted by false discovery rate (FDR). Significance was assumed at a *P* value of < 0.05. Data were expressed as means ± standard errors of the means (SEM).
