# Supplementary figures and images for: Integrated Role of Bifidobacterium animalis subsp. lactis Supplementation in Gut Microbiota, Immunity, and Metabolism of Infant Rhesus Monkeys
Source: mSystems. 2016 Nov 29;1(6):e00128-16. doi: 10.1128/mSystems.00128-16 (PMC5128019; doi:10.1128/mSystems.00128-16)

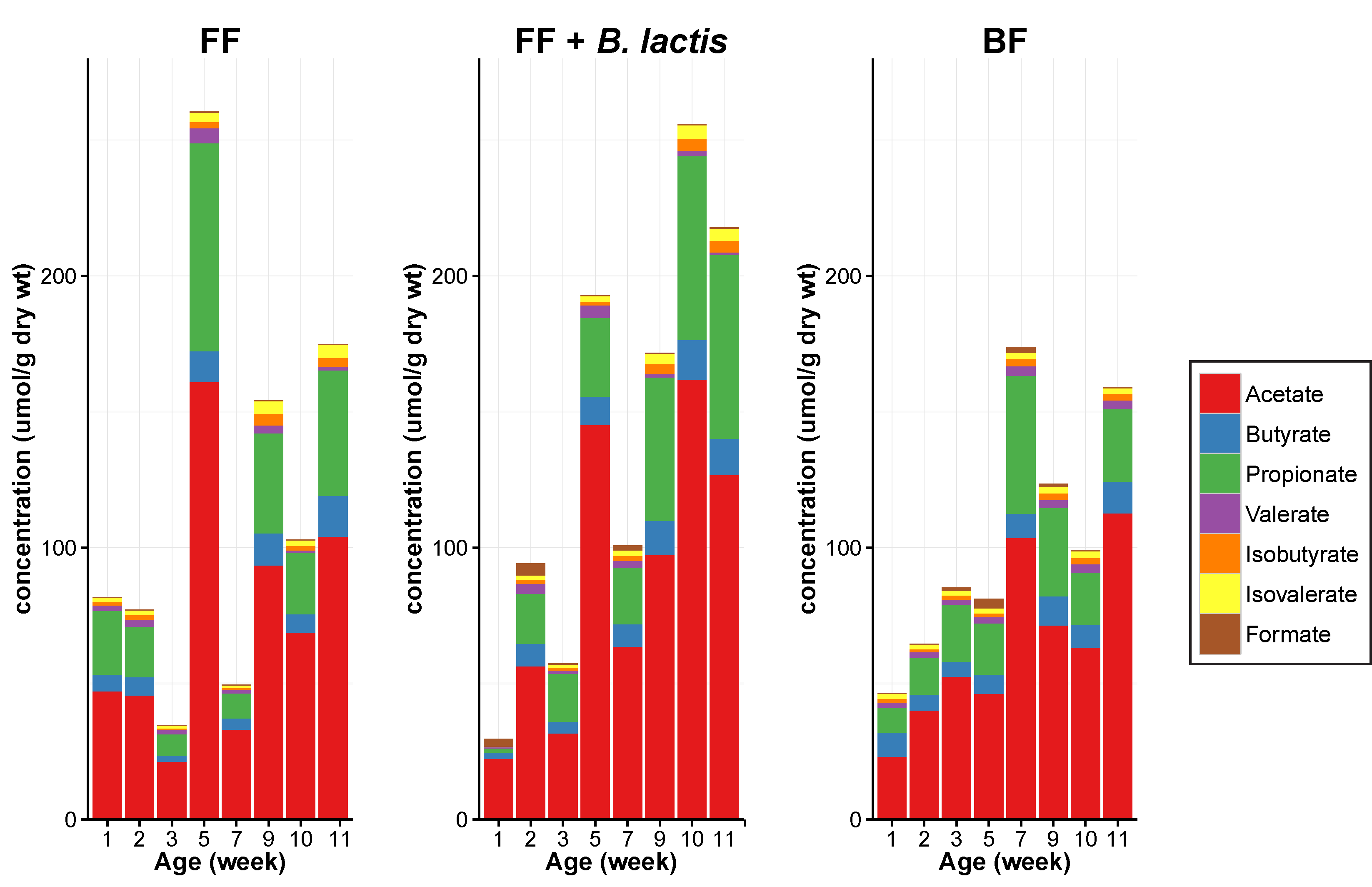

Supplement: Figure S1 [file sys006162064sf1.tif]
